# Complications and Survivorship of Distal Humeral Allograft Reconstruction After Tumor Resection: Literature Review and Case Series

**DOI:** 10.5435/JAAOSGlobal-D-20-00256

**Published:** 2021-02-11

**Authors:** Pramod Kamalapathy, Akash Shah, Kevin Raskin, Joseph H. Schwab, Santiago A. Lozano-Calderón

**Affiliations:** From Department of Orthopedic Surgery, Division of Musculoskeletal Oncology, Massachusetts General Hospital Cancer Center, Massachussetts General Hospital, Boston, MA.

## Abstract

Primary bone sarcomas of the proximal humerus represent most bone sarcomas in the upper extremity. However, limited literature exists on the survival and complications of distal humeral allograft reconstruction because of the relative rarity of disease at the distal end.

**Methods:**

We performed a literature review in PubMed and EMBASE using the terms“Humerus,””Distal,” “Allograft,” and “Reconstruction” to include all the patients with distal humeral reconstructions. In addition, a retrospective review of all patients who underwent distal humerus tumor resection and allograft reconstruction at our tertiary care center over a 23-year period was performed.

**Results:**

Fourteen patients in four different case series have been reported previously with an allograft survival rate of 86%. Thirty-six percent of patients experienced complications, most commonly nonunion (3) and allograft subluxation (2). In a retrospective review at our institution, six met the inclusion criteria and were found to have similar complications.

**Conclusion:**

Based on our experience and the review of the literature, nonunions are the most frequent complication and junctional fractures are the most common cause of revision of allografts in this location. Larger sample studies are required to identify potential correctable predictors of these complications. In addition, complications should be correlated with functional outcome data.

Primary malignant bone tumors account for 0.8% to 1% of all malignancies in the body, with approximately 2,800 new cases diagnosed each year in the United States.^1,2^ Over the past 30 years, allograft reconstruction has gained popularity for use in large skeletal defects throughout the body because of its ability to preserve bone stock.^3,4,5,6,7,8,9,10,11^ The distal humerus is an uncommon site for primary malignant bone tumors or metastatic disease. It is also a location near important neurovascular structures in the upper extremity. Accordingly, surgical treatment of tumors in this location represents a challenging task. Reconstructive options include arthrodesis, endoprostheses, resection arthroplasty, and allograft reconstruction.^6,12,13,14,15,16^ In treating young, active patients, reconstruction can afford improved functional outcomes and cosmesis, although implant or allograft survival depends on multiple factors.^7,9-11,18,19^

Complications from allograft reconstruction include infection, subluxation, fracture, and nonunion. Megaprostheses present similar complications in addition to aseptic or septic loosening.^9-11,17,18,19,20,21,22,23,24^ The current literature on the complications of osteoarticular allograft reconstruction of distal humerus tumors is limited and underpowered, given the rare occurrence of tumors in this location. The challenges of obtaining local tumor control and subsequent reconstruction are substantiated by the close proximity of vital neurovascular structures and poor soft-tissue coverage. The purposes of this study after reviewing the published literature and our institution's experience were to determine the complications associated with distal humeral osteoarticular allograft (DOA) reconstruction, their rate of survival, and mechanisms of failure.

## Study Questions

1. What are the complications associated with DOA reconstruction?
2. What is the survival rate of distal humerus osteoarticular allografts?
3. What are the failure mechanism of distal humerus osteoarticular allografts?

## Methods

### Literature Review

We performed a literature review in PubMed and EMBASE using the terms “Humerus,”“Distal,”“Allograft,”and “Reconstruction.”After cross-referencing articles and references, we included all the patients with distal humeral reconstructions that are published in the literature. A total of 14 patients from case series and case reports were retrieved for analysis. Several small series are reported in the literature which have assessed the complications of distal humerus osteoarticular allografts (Literature Review Table, Supplemental Digital Content 1, http://links.lww.com/JG9/A110). Each of these studies have DOA reconstruction as a subset of larger study cohorts or case reports. No study to date exists, which has only analyzed a large case series of DOA reconstructions.

### Institutional Case Series

After institutional review board approval, a retrospective review of prospectively collected data was performed for a consecutive series of primary malignant bone tumors of the distal humerus treated by fellowship-trained orthopaedic oncologists at our academic tertiary care center over a 23-year period.

Inclusion criteria included skeletally mature patients with a primary malignant or metastatic bone lesion of the distal humerus without previous surgery to the extremity. Exclusion criteria included skeletally immature patients. Of the seven patients screened, six met the inclusion criteria. This group became our study cohort. One patient was excluded because of lack of follow-up after surgery in our institution. After surgery, the patient continued his orthopaedic care locally because he lived out of the state.

#### Surgical Perioperative Care

Drains were used in all six patients and were removed postoperatively when output was deemed minimal by the treating surgeon (<30 cc/d). All patients were treated prophylactically with oral antibiotics (first-generation cephalosporins) for a period of at least 1 month. The earliest cases were treated with oral antibiotics for 3 months. All patients were kept nonweightbearing for a period of 8 to 10 weeks. Physical therapy with early active/assisted and passive range of motion was started in all patients within 2 weeks of surgery while immobilized in an articulated hinged elbow brace.

Patients with primary malignant tumors were seen at regular follow-up intervals of 2, 6, and 12 weeks and every 3 months thereafter for 2 years, every 6 months for the subsequent 3 years, and annually for the following 5 years. At the initial three follow-up visits, all patients were evaluated with AP and lateral views of the treated elbow. Oncologic follow-up with imaging of the chest (alternating chest radiograph and chest CT) and affected elbow radiographs AP and lateral views were performed thereafter. If any changes were visualized on radiographs or changes were seen on physical examination, additional axial imaging with CT scan or MRI was obtained. The patient with metastatic disease was followed at 2, 6, and 12 weeks and every 6 months thereafter until death. In this patient, the first three visits were also only elbow radiographs, followed by CT scans of chest, abdomen, and pelvis in combination of elbow radiographs every 6 months thereafter.

The distal humerus was defined as the distal one-third of the total length of the humerus. The stage of the tumor was determined preoperatively using radiographs, CT scans, and/or MRI. The grade of the tumor was based on core biopsy results, which served as an indication for resection. Negative margins after tumor removal were confirmed by intraoperative biopsy of the remaining bone marrow (early cases) and by macroscopic evaluation of the specimen before allograft reconstruction (late cases). After tumor removal, the size of the tumor was measured intraoperatively using a sterile ruler. Cadaveric fresh-frozen osteoarticular allografts were used in all patients and were obtained by our institution's bone bank. The size of the allograft used in reconstruction was determined by the surgeon's preference and measured using the same preoperative technique.

#### Surgical Technique

For surgical technique, earlier cases were approached anteromedially with the patient in the supine position, especially if there was a soft-tissue mass component extending anteromedially or medially near the arm's neurovascular bundle. The incision is done usually starting in the medial arm for identification and dissection on the brachial artery and veins as well as the median and ulnar nerves. Through this window, the medial brachial septum can be dissected, given access to the anterior and medial compartments of the arm. The approach is extended distally by curving the incision into the forearm anteriorly to the medial epicondyle. The incision is extended as a medial or an anteromedial approach to the forearm. The use of the direct anterior approach was also very common early on in our Institution's practice. This approach is started through a transverse incision in the elbow crease, extending proximally as the medial approach to the arm and distally as the anterolateral or Henry approach into the forearm. In many occasions, a posterior approach was additionally used for better soft-tissue coverage and better positioning of the hardware, being plates of our preference. More points of distal fixation are attainable in the distal portion of the humerus allograft with constructs fixed posteriorly than anteriorly. After the resection and allograft placement, the ulnar collateral ligament and radial collateral ligaments were reattached to native structures.

Before the common use of the Gerwin's approach,^25^ the common practice for the additional posterior approach was the direct posterior approach with the olecranon chevron osteotomy for better visualization of the elbow joint. Our current preference is to do most of the dissection and resection posteriorly through the Gerwin approach, with identification and mobilization of the radial nerve through the inferior lateral cutaneous branch of the radial nerve. An early diaphyseal osteotomy of the humerus allows mobilization of the distal humerus, facilitating tumor excision and preservation of the medial structures. If necessary, an additional medial approach to the arm can be used for identification, dissection, and protection of the brachial artery and veins as well as the median and ulnar nerves. This additional medial approach is smaller in length and less frequently used in recent years, given the versatility and field of exposure attainable with the Gerwin's approach. Another advantage of mobilizing the lateral head of the triceps through this approach is avoiding the olecranon osteotomy.

Finally, our preferred method of fixation is plates and screws. For all patients in this case series, we used two dynamic compression plates and most commonly four screws on either side (Institutional Case Series Surgical and follow up Information, Supplemental Digital Content 1, http://links.lww.com/JG9/A110). All plates were fixed to the allograft and humeral shaft. For one patient, two plates were placed lateral and medial to the site of osteosynthesis. No intermedullary rods were used in the fixation for these patients. Early on, with the use of nonlocking plates, we observed higher rates of nonunion than with the subsequent locking plates. This is an observation from the fixation of allografts in other locations in the upper extremity such as the proximal humerus, the humeral and radial diaphysis, and the distal radius. We believe that the distal humerus is not an exception to the rule, and fixation with periarticular locking plates should provide better allograft integration rates than nonlocking plate.^26,27^ In the past, medial plates were used. We disfavor their use, given the lack of points of fixation distally in the allograft. Our preference is the use of the distal humerus periarticular and extra-articular distal humerus plate or reconstruction with bicolumnar distal locking periarticular humerus plates. We prefer the former because of its larger size and length. At least six cortical points of fixation should be achieved in the host bone and the allograft (three bicortical screws or a combination of bicortical and unicortical screws), providing these six points of fixation. Intramedullary rods have been used because fracture of the allograft is also a concern. However, the length of the rod and fixation distally is not sufficient to span the entirety of the allograft. If a rod is used, additional plate should be added for fixation to improve rotation control and increase points of fixation distally with locking screws. The use of vascularized fibula autograft in our practice is a rescue procedure used to supplement nonunited or delayed union allograft/host interfaces or to treat nondisplaced fractures of the allograft.

Owing to the small size of this series, only descriptive statistics were applied. The same modality was used to summarize the data in the published literature. Statistical analyses were performed with STATA 13.0 (StataCorp LP).

## Results

### Literature Review

Four articles reviewed oncologic patients undergoing distal humerus reconstruction (Literature Review Table, Supplemental Digital Content 2, http://links.lww.com/JG9/A111). A total of 14 patients were identified. The mean patient age was 34 years old (range, 14 to 66). Tumors included giant cell bone tumor, solitary skeletal metastases, chondrosarcoma, fibrosarcoma, malignant myxoid epithelioid tumor, Ewing sarcoma, lymphoma, and metastatic renal cell carcinoma. The indications for surgery are listed in Supplemental Digital Content 2 (Literature Review Table, Supplemental Digital Content 2, http://links.lww.com/JG9/A111). The average survival was 86% at 5 years, and the average complication rate was 36%. Of the 14 patients found in the literature, there were 5 (36%) patients who experienced complications. These case series have been summarized in Supplemental Digital Content 2 (Literature Review Table, Supplemental Digital Content 2, http://links.lww.com/JG9/A111). The most common complication was nonunion (3, 21%). Infections (1, 7%), subluxation (1, 7%), and radial nerve palsy (1, 7%) were less frequent complications.

Kharrazi et al treated 18 consecutive patients with 16 hemicondylar allografts and three total elbow osteoarticular allografts (distal humerus and proximal ulna). Tumors included six giant cell bone tumors, five chondrosarcomas, a fibrosarcoma, a malignant myxoid epithelial tumor, a malignant fibrous histiocytoma of bone, an Ewing sarcoma, a lymphoma, a chondromyxoid fibroma, and a metastatic renal cell carcinoma. With an average follow-up length of 102 months, their reported survival rate at the final follow-up was 82%. The overall complication rate was 27% with one infection, one dislocation, and one nonunion. There was one superficial infection that resolved after empiric antibiotics. The dislocation occurred one month after surgery because of a fall and was treated by closed reduction. The nonunion was declared at 6 months after surgery, but the patient was reported to be pain-free and with intact internal fixation at his last follow-up.^9^

Aponte-Tinao et al^28^ had one patient who underwent DOA reconstruction in their study. During the 60-month follow-up, the patient developed a nonunion, which was treated with revision open reduction and internal fixation (ORIF) with autologous bone graft.

Gasbarrini et al^29^ presented a case report on a 42-year-old man with an aggressive giant cell tumor. No complications were noted in their follow-up of 72 months. Unlike this study, however, the patient underwent partial DOA reconstruction for a giant cell tumor in which some of the host bone and articular surface was retained.

Fernández-Valencia et al^30^ reported on one patient who underwent DOA for metastatic breast cancer. The patient was a 48-year-old woman with a solitary skeletal metastasis in the distal left humerus. During the 11-year follow-up, the patient had radial nerve neuroapraxia, which completely resolved at 2 months with nonsurgical management. In addition, the patient progressed to nonunion at 9 months and underwent revision ORIF with autologous bone grafting. Six months after the second operation, the patient achieved union.

### Institutional Case Series

In our case series, three patients were men and three were women. Patient ages ranged from 18 to 69 with a mean age of 37 years. Patient baseline characteristics, tumor type, and the use of adjuvant therapy before or subsequent to reconstruction are included in Supplemental Digital Content 3 (Institutional Case Series Table, Supplemental Digital Content 3, http://links.lww.com/JG9/A112). Tumors included 2 (33%) chondrosarcomas, 2 (33%) Ewing sarcomas, 1 (17%) metastatic lesion from a primary GI tumor, and 1 (17%) primary lymphoma of bone. Of the seven eligible patients to participate in the study, one was excluded because of oncologic follow-up at another institution after completion of surgery. No information was available for that patient.

**Figure 1:**
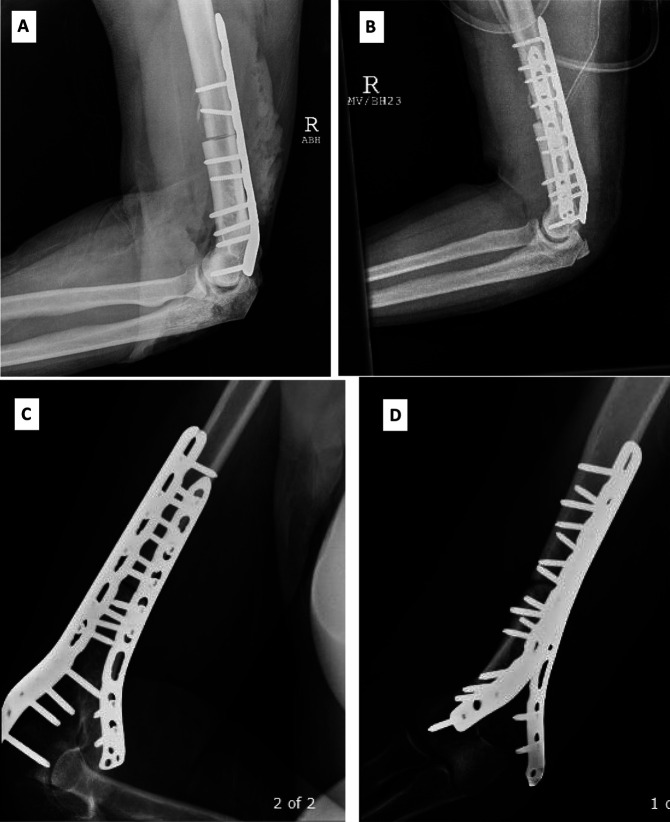
Photographs of the patient who experienced nonunion. **A** and **B**, Images during surgery. **C** and **D**, Photographs 12 months before revision surgery.

The most common complication among our patients was allograft subluxation (2), and the most common reason for revision surgery was allograft junctional fracture (2). Nonunion (1) and radial nerve palsy (1) were less frequent complications. Images of patient who experienced nonunion is depicted in Figure 1. There were no infections. The two patients with allograft fractures sustained them at 108 and 24 months (9 years and 2 years) after the index distal humerus osteoarticular reconstruction. Both patients with allograft fractures underwent revision surgery with ORIF with iliac crest bone autograft and had no further complications. Both fractures were located near the host bone/allograft junction. No revision, removal, or exchange of the allograft was necessary. The nonunion patient underwent revision of the osteosynthesis 12 months after the index procedure with autologous bone graft and had no further complications (Institutional Case Series Table, Supplemental Digital Content 2, http://links.lww.com/JG9/A111).

The mean follow-up interval for our study cohort was 69.5 months (range, 5 to 249 months). The rate of allograft survival was 83%, with one patient who died because of systemic progression of disease. Of the remaining five patients, one patient had tumor local recurrence, which was excised successfully without further recurrence. No allograft failed because of infection. Patients at the postoperative follow-up noted comparable nerve function with preoperative assessment and demonstrated no pain. One patient was found to have a radial nerve injury because of a second surgery, and another patient had consistent pain because of recurrent lymphoma. All patient had extension lag of the elbow at some extend (Institutional Case Series Surgical and follow up Information, Supplemental Digital Content 1, http://links.lww.com/JG9/A110).

Only one patient reported functional outcome data: QuickDASH: 4.5, QuickDASH Optional Work Module: 0, PROMIS Physical Function Upper Extremity: 78, PROMIS Pain Interference: 9, and TESS Upper Extremity: 98.7. The other patients did not participate in the study by filling function questionnaires becayse they were unable to be reached or did not want to participate.

## Discussion

TheAanagement of tumors of the distal humerus with resection and allograft reconstruction is a challenging task, which emphasizes the difficult balance between complete tumor removal and maximum functional outcome. As the knowledge of tumor biology and chemotherapy agents improve, the possibility for implementing a more durable limb-salvaging reconstruction becomes more feasible. Several studies have shown the success of allograft reconstruction for tumors of the proximal humerus;^7,10-12,18,19,31^ however, limited literature for DOA reconstruction exists.

We found in this study that nonunion is the most common complication. The second-most frequent complication is allograft fracture, which is a consistently prevalent event in allograft reconstruction of the proximal humerus in addition to subluxation.^32^ Other complications from allograft reconstruction of the distal humerus include nerve injury and infections. Contrary to the common perception of the high risk of infection in this location because of soft-tissue coverage challenges, infection was not relevant. In fact, we did not encounter any infections in our institutional case series.

In the literature review, DOA reconstruction survival was 83%, with nonunion being the most common complication. Our rate of allograft survival was similar to the large case series by Kharrazi et al.^9^ However, our infection rate was lower, 0% and 9% respectively.^9^ In addition, we had two patients with junctional fractures (33%), whereas the literature review did not yield any fracture complications. Moreover, complications in our study cohort occurred much later after the index DOA reconstruction, between 12 to 108 months, than that reported in the literature, between 6 to 9 months.^9,28-30,33^ These results could be because of a number of factors including patient age, comorbidities, bone stock, tumor type, the size of the allograft, previous functional activity, postoperative rehabilitation regimens, and the length of the follow-up.^7,8,9,10,11,17-19,22^ Overall, in comparing existing studies of DOA reconstruction, our large case series had a longer follow-up interval and reported fewer complications.

Although infection may be perceived as the leading complication, Meijer et al showed that infection rates for osteoarticular allografts were similar to endoprostheses and allograft-prosthesis composites for proximal humerus reconstruction.^30^ This study gives further evidence that infection does not seem to be a notable cause of failure in the humerus, as it is described with allografts in general. Our group reported a 12% infection rate in 150 patients who underwent proximal humerus reconstruction, with no difference between osteoarticular allograft, endoprostheses, and allograft-prosthetic composites.^34^ We cited low preoperative hemoglobin and albumin levels as independent risk factors for infection in that patient population.^34^ Lord et al^22^ reported an 11.7% infection rate in their allograft cohort. Cases with more extensive surgery including loss of bone, soft tissue, or skin had a higher infection risk.^22^

Furthermore, several studies have shown allograft fracture, and not infection, to be the most common cause of failure and revision surgery in proximal humerus allograft reconstruction.^31,32^ It is also important to note that fractures were the most common reason for revision surgery in our case series but were not found in the published literature. It is commonly thought that fractures are the number one reason for failure and subsequent revision surgery in allograft reconstructions, but this study shows that it is also important to assess nonunion and joint instability risk factors as well to prevent complications. It is important to recognize the high revision surgery rate to retain the allograft, which some argue are part of the process of achieving allograft retention in the long term.

Several limitations to this study exist. First, the retrospective nature and small sample size limit the generalizability of the results, but they do provide a broad understanding of the feasibility and acceptable survival rate from allograft reconstruction. Moreover, we were unable to correlate our findings with functional scoring as evidenced by our one responder. Second, tumors in the study included primary bone tumors, metastatic lesions, and lymphoma—all of which have different cellular biology and can alter complication rates. However, we believe this myriad of tumors represents a realistic problem encountered in an active orthopaedic oncology practice. Third, two of six of our patients were lost before two years, so complication frequency may in fact be higher than estimated. Nevertheless, this is the largest case series and revision of the literature of patients with distal humerus osteoarticular allorgrafts. Nowadays, reconstruction of the distal humerus is more commonly done with megaprostheses such as distal humeral replacements, given more predictable functional results. The use of these prostheses is limited in the pediatric population in which it is common to use DOA reconstructions because of the preservation of both bone stock and the proximal ulna epiphyseal plate. It is important to know this information about distal humeral allografts and have it available to the orthopedic oncology community, with the purposes of inform and counsel patients appropriately while discussing allograft survival rates, function, complications and modes of failure as allograft reconstruction of the distal humerus still has a role in the young and the young adult patient.

## Conclusion

Our analysis of the complications associated with the DOA reconstruction and factors associated with survival provides greater insight into the lifespan of limb-salvaging options after tumor resection of the distal humerus. DOA reconstruction demonstrates a durable limb-salvaging solution in young, physically active patients with an acceptable complication rate. Contrary to what is commonly believed, our experience and the data published in the literature demonstrate that infection is not a common complication in allograft reconstruction of the distal humerus. Based on our experience and the review of the literature, nonunions are the most frequent complication and junctional fractures are the most common reason for revision surgery.

## Supplementary Material

SUPPLEMENTARY MATERIAL
