# Supplementary material for: Complications and Survivorship of Distal Humeral Allograft Reconstruction After Tumor Resection: Literature Review and Case Series
Source: J Am Acad Orthop Surg Glob Res Rev. 2021 Feb 11;5(2):e20.00256. doi: 10.5435/JAAOSGlobal-D-20-00256 (PMC7886443; doi:10.5435/JAAOSGlobal-D-20-00256)
Supplement: SUPPLEMENTARY MATERIAL [file jagrr-5-e20.00256-s001.docx]

Supplemental Table 1. Institutional Case Series Surgical and follow up Information

| Patient | DC Plate | Screws on each side of osteosynthesis site | Resection  Length (cm) | Pain* | Pain at last F/u | Nerve function* | Nerve function at last f/u | Range of motion at last follow-up |
| --- | --- | --- | --- | --- | --- | --- | --- | --- |
| 1 | 8 hole | 3 | 23 | Moderate pain | No pain | Normal | Radial Nerve injury d/t surgery; otherwise normal | Flex: 110 deg Ext: -25 deg |
| 2 | 8 hole | 3 | 19 | Severe pain | Pain d/t recurrent lymphoma | Normal | Normal | Flex: 90 deg Ext:-30 deg |
| 3 | 8 hole | 4 | 20 | Severe pain | Mild pain | Normal | Normal | Flex: 110 deg. Ext: 15 deg |
| 4 | 8 hole | 4 | 15 | Severe pain | Mild pain | Slight decrease | Normal | Flex: 125 deg. Ext: -30 deg. |
| 5 | 8 hole | All screws except distal | 14 | Moderate pain | No pain | Normal | Normal | Flex: 120 deg.  Ext: -10 deg |
| 6 | 10 hole (lateral): Screws in every hold except distal  9 hole (medial): one non locking on either side | | 8.5 | None | No pain | Normal | Normal | Flex: 125 deg  Ext: -15 deg |

*Denotes prior to surgery

d/t = due to
