# Supplementary material for: Complications and Survivorship of Distal Humeral Allograft Reconstruction After Tumor Resection: Literature Review and Case Series
Source: J Am Acad Orthop Surg Glob Res Rev. 2021 Feb 11;5(2):e20.00256. doi: 10.5435/JAAOSGlobal-D-20-00256 (PMC7886443; doi:10.5435/JAAOSGlobal-D-20-00256)
Supplement: SUPPLEMENTARY MATERIAL [file jagrr-5-e20.00256-s002.docx]

Supplemental Table 2. Summary of Literature Review

| Authors | Kharrazi et al. | Aponte-Tinao et al. | Gasbarrini et al. | Fernandez-Valencia et al. |
| --- | --- | --- | --- | --- |
| Year | 2008 | 2013 | 2008 | 2002 |
| Number of Patients | 11 | 1 | 1 | 1 |
| Sex | 10 female,9 male* | 38 men, 32 woman** | Male | Woman |
| Age | 32 | 32 | 42 | 48 |
| Histology | *Giant cell tumors, chondrosarcomas, fibrosarcoma, malignant myxoid epithelial tumor, malignant fibrous histiocytoma of bone, Ewing’s sarcoma, lymphoma, chondromyxoid fibroma, metastatic renal cell carcinoma | **Unknown | Giant Cell Tumor | Solitary skeletal metastasis |
| Survival Rate at Followup (%) | 82% | 100% | 100% | 100% |
| Follow-up (months) | 102 | 60 | 72 | 132 |
| Infection (%) | 1 (9%) | 0 | 0 | 0 |
| Fracture (%) | 0 | 0 | 0 | 0 |
| Joint Instability (sublux, disloc) (%) | 1 (9%) (disloc) | 0 | 0 | 0 |
| Nonunion (%) | 1 (9%) | 1 (100%) | 0 | 1 (100%) |
| Nerve injury (%) | 0 | 0 | 0 | 1 (100%) |
| Complication Rate (%) | 27% | 100% | 0 | 100% |

*Series contained 18 patients, among which 11 patients had distal humeral reconstructions

**Series contains 38 men and 32 woman, among which 1 patient had distal humerus reconstruction
