# Supplementary material for: Complications and Survivorship of Distal Humeral Allograft Reconstruction After Tumor Resection: Literature Review and Case Series
Source: J Am Acad Orthop Surg Glob Res Rev. 2021 Feb 11;5(2):e20.00256. doi: 10.5435/JAAOSGlobal-D-20-00256 (PMC7886443; doi:10.5435/JAAOSGlobal-D-20-00256)
Supplement: SUPPLEMENTARY MATERIAL [file jagrr-5-e20.00256-s003.docx]

Supplemental Table 3. Institutional Case Series

| Patient | Age | Sex | Diagnosis | Smoker | Follow up (months) | Outcome | Complication | Adjuvant Tx |
| --- | --- | --- | --- | --- | --- | --- | --- | --- |
| 1 | 18 | F | Ewing Sarcoma | N | 33 | Tumor Recurrence (excisional biopsy) | Nerve injury | Chemotherapy and Radiation Tx |
| 2 | 31 | F | Lymphoma | N | 18 | Tumor Recurrence (no reop), death | None | Chemotherapy |
| 3 | 36 | M | Chondrosarcoma | Y | 249 | Fracture (s/p ORIF) | Fracture | None |
| 4 | 69 | M | Metastasis (GI tumor) | Y | 5 | Subluxation (no reop) | Subluxation | None |
| 5 | 37 | M | Chondrosarcoma | Y | 37 | Subluxation and fracture (s/p ORIF) | Fracture/subluxation | None |
| 6 | 34 | F | Ewing Sarcoma | Y | 75 | Nonunion (s/p revision) | Nonunion | Chemotherapy |
